# Effect of Self-regulation Training on Management of Type 2 Diabetes

**DOI:** 10.5812/ircmj.13506

**Published:** 2014-04-05

**Authors:** Jahanshir Tavakolizadeh, Mehri Moghadas, Hami Ashraf

**Affiliations:** 1 Department of Basic Medical Sciences, Social Development and Health Promotion Research Center, Gonabad University of Medical Sciences, Gonabad, IR Iran; 2 Health and Treatment Network of Bajestan, Gonabad University of Medical Sciences, Gonabad, IR Iran; 3 Department of Research and Education, Razavi Hospital, Mashhad, IR Iran

**Keywords:** Diabetes Mellitus, Type 2, Nutritional Sciences, Motor Activity

## Abstract

**Background:**

Diabetes is the most common metabolic disorder which is required to be taken under control. According to some studies, the impact of self-regulation on health has been considered as a monitoring strategy to achieve individual’s goals.

**Objectives:**

This study, which was performed in 2012, aimed at determining the consequences of self-regulation on controlling type 2 diabetes.

**Patients and Methods:**

In this double-blind clinical trial, 60 patients with type 2 diabetes - who were referred to Diabetes Clinic of Bisto-Dou Bahman Hospital in Gonabad, Iran - were divided randomly into case and control groups. Self-regulation questionnaire, nutritional information and physical activity checklists were completed by both groups once at the beginning and once at end of the training. Fasting blood sugar (FBS) of both groups were measured as well. Case group was trained for a month, whereas the control group received no special training. Data were analyzed by SPSS version 19 software, K-square and paired t-tests.

**Results:**

Comparing the case with the control group before and after the training showed that teaching patients self-regulatory strategies had significant impact on lowering blood sugar (-16.50 vs. -2.47, P < 0.001), observing dietary behaviors (5.97 vs. -0.87, P < 0.001) and increasing physical activities (6.2 vs. -0.73, P < 0.001) of the former group.

**Conclusions:**

Learning self-regulations has a role to play in controlling type 2 diabetes. Therefore, it is suggested to professionals in healthcare centers to educate patients about self-regulatory techniques and use them as auxiliary methods for keeping type 2 diabetes under control.

## 1. Background

Type 2 diabetes is a metabolic disorder caused by insulin resistance and relative reduction of insulin production. Insulin resistance is defined as the diminished ability of cells or tissues to respond to the physiological concentrations of insulin (1). According to World Health Organization (WHO), prevalence of type 2 diabetes will have been increasing from 2.8 percent (177 million people) in 2000 to 4.4 percent (366 million people) by 2030 (2). According to cognitive-pandemic studies, prevalence rate of diabetes in Iran has been reported between 5% and 8%. Notably, 90% of the patients have diabetes type 2 and the other 10% have diabetes type 1. It is worth pointing out that nearly half the diabetic patients are not aware of their condition (3). In this regard once type 2 diabetes is diagnosed, it is expected that patients themselves take steps to regulate their disease. Such self-regulated learning skills include symptom management, treatment continuum, maintenance of healthy life style and daily control of complications. Regardless the severity of the disease, self-regulated learning also plays a key role in patients’ survival (4).

Given the nature of the disease and its complications, it appears that self-regulated learning is useful for people diagnosed with diabetes. Despite the importance of the disease prevention and the treatment, namely providing specific treatment strategies, there exist limited research on diabetes medication adherence. On the other hand, the findings on the decision making process in self-regulation training with regard to diabetes are not adequate (5). Fundamental structures of self-regulation learning skills involve the processes of goal-setting, planning to achieve the goals, monitoring them, comparing them with standards and changing the behaviors to improve performance (6). There are several major self-regulation models such as Pintrich and De Groot (7), Zimmerman (8), Carver & Scheier (9) and Bandura (10); however, it was Bandura self-regulation learning method which was employed in this study. Bandura believed that self-regulated training is the interaction between three components: self-observation, self-judgment and self-response, identified as:

- Self-observation: regular attention to the individual performance, self-proving and a factor to reach the goals,
- Self-judgment: regular comparison of individual performance with the existing standards and goals,
- Self-response: creating meaningful changes to achieve a goal (10, 11).

In his paper, Bandura pointed out that human beings observe divergent trends in health issues. Enormous resources are used to eliminate bad health habits. The conception of health is changing from a disease model to a health model. This model emphasizes more on health promotion rather than disease management. Bandura claims that health quality is deeply influenced by lifestyle habits and self-management (10). Notably, such changes are not possible without self-regulatory learning skills and its components. Various studies have been performed on self-regulatory skills along with trainings about physical and mental health. Some of these studies include self-regulated training and mental health (12), smoking (13), chronic pain (14), bulimia nervosa and regulatory disorder (15). In addition, some correlative studies have also been conducted on self-regulation training and diabetes (12, 16-18). In a research recently conducted on self-regulation training and diabetes, patients compared their condition with standard ones. When blood sugar was high, they did some certain physical activities to lower the blood sugar and reduce the symptoms. According to the findings, there was a significant relation among using insulin, disease symptoms and physical activities; however, no considerable relation was observed between metabolic control and the related symptoms (19). In another study, it was revealed that self-management had slight impacts on behavior change in diabetes. In this study, which proceeded for a year, self-management only resulted in certain modifications in FBS and Hemoglobin (20).

In Iran, similar studies have been performed in terms of the relation among self-regulated learning, eating attitudes and life style (21), effective factors on self-regulation of hypertension (22) and the impact of self-management on blood sugar control (23). The outcome of nutrition and walking trainings on controlling blood sugar (24) and the effect of dietary changes on FBS and body mass index (BMI) in patients with type 2 diabetes (25) have been also investigated in some other studies. The objectives the educational intervention programs are to examine how diabetics can be prevented, treated and controlled in a way that its complications be avoided more effectively (26).

## 2. Objectives

This study in particular aimed at evaluating the effectiveness of self-regulation training to manage type 2 diabetes by focusing on 3 variables: lowering blood sugar, diet-observing and increasing physical activities in diabetic patients.

## 3. Materials and Methods

The case under study was a double-blind clinical trial. One thousand, five hundred and sixty patients with type 2 diabetes - who were referred to Diabetes Clinic of Bisto-Dou Bahman Hospital (a general referral governmental hospital, with seven wards and 115 beds) in Gonabad, Iran - were included in this study during the year 2012. Samples were selected from patients who had diabetes for more than 4 years as well as those who participated in training sessions. The following patients were excluded from the study:

- Those who were not interested in participating in the study,
- Those who faced traumatic life events during the study such as loss of a close relative,
- Those who had major problems such as illness or living in a different place which make them not being able to participate in the training courses.

The sample size was calculated through the same method employed by Brown et al. in 2002 (20). Regarding fasten blood sugar difference between the two groups (37.52 mg/dL) with 95% confidence and the 80% power, the sample size was evaluated by comparing the means. Each group comprised of 30 patients, which make the sample size 60 in total. No patient was excluded and no data missed during the study.

### 3.1. Random Allocation

All the patients included in the study were registered in Diabetes Clinic of Bisto-Dou Bahman Hospital, Gonabad, Iran and they all had registration numbers. By using random allocation software, 30 patients were indiscriminately assigned to the case and 30 to the control group.

### 3.2. Intervention and Equipment

Self-regulation questionnaire, nutritional information and physical activity checklists were completed by both groups. FBS were measured by one auto-analyzer BT3000 twice for both groups once at the beginning and once at the end of the study, i. e. before and after the training program (27). The questionnaires were completed under the supervision of the research worker. All the instruments in the clinic were calibrated routinely. The case group received self-regulation. Ten sessions of 65 minutes were held for a month time in the form of direct instruction including lecture and group Q & A discussion. Bandura’s model was applied to teach self-regulatory training strategies to the case group. The training was based on self-observation, self-assessment, self-reflection (including behavioral, personal and environmental self-reflection) and making plans to achieve the goals. Training sessions were held in Diabetes Clinic of Bisto-Dou Bahman Hospital in Gonabad, Iran and self-regulated training was provided by clinical psychologists. Control group received no special training. No patient withdrew from the program and all the patients completed the treatment protocol. There was no missing data.

### 3.3. Statistics

Data were analyzed by SPSS version 19 software, K-square, paired t-test and student's t-test were used to test normality of the distribution which then approved by Kolmogroph-Smirnov test. In 2004, Carey, Neal and Collins made some changes to the self-regulation questionnaire (SRQ) (63 items) . The new SRQ was a 31-item questionnaire and the scoring method was designed with respect to five-point Likert scale (27). This questionnaire correlated strongly with the original form and indicated to have a good internal consistency. Furthermore, the reliability of the test was determined at 0.94 (28). Health promoting lifestyle questionnaires were also used to assess the effects of physical activity and diet-observing on self-regulation (29). The multiple choice questionnaire included 52 items on the six aspects of health-promoting behaviors, comprising nutrition, physical activity, spiritual growth, health responsibility, stress management and interpersonal relations (30). In this study, all items had acceptable item-total correlations (P > 0.34). Test-retest results showed stability for not only HPLPII but also subscales. The confirmatory factor analysis related to six-factor model represented an acceptable fit. Mohammadi Zeidi et al. reported an acceptable validity and reliability for the Persian version of the questionnaire (30) which was employed for the purposes of this study.

## 4. Results

The mean age of the case group was 58.93 ± 11.12 and 54.53 ± 9.06 for the control group. Mean duration of the disease was reported 7.63 ± 3.82 years in case group and 6.16 ± 2.13 years in control group. According to the outcome evaluated by Mann-Whitney and Chi-square tests, both groups had the same education level. Moreover, no significant difference was observed between the case and control groups in terms of demographic factors (Table 1). The results of t-test (independent samples t-test) were calculated based on the differential between means of the self-regulations with regard to a pretest and posttest taken from case and control groups (first P value) (Table 2). As a result, mean of self-regulation training was higher in case than the control group (P = 0.001).

**Table 1.** Distribution of Demographic Factors in Case and Control Groups ^a^

Demographic Factors	Case	Control Group	Total	P Value
**Gender**				1
Male	20 (66.7)	20 (66.7)	40 (66.7)	
Female	10 (33.3)	10 (33.3)	20 (33.3)	
Total	30 (100)	30 (100)	60 (100)	
**Marital status**				0.875
Married	20 (66.7)	19 (63.3)	39 (65)	
Single	2 (6.7)	1 (3.3)	3 (5)	
Died	4 (13.3)	8 (26.7)	12 (20)	
Divorced	4 (13.3)	2 (6.7)	6 (10)	
Total	30 (100)	30 (100)	60 (100)	
**Education level**				0.642
Illiterate	2 (6.7)	1 (3.3)	3 (5)	
Elementary school	7 (23.3)	5 (16.7)	12 (25)	
Guidance school	7 (23.3)	8 (26.7)	15 (25)	
High school	5 (16.7)	8 (26.7)	13 (21.7)	
Higher education	9 (30)	8 (26.7)	17 (28.3)	
Total	30 (100)	30 (100)	60 (100)	
**Diabetics in family**				0.797
Yes	16 (53.3)	17 (56.7)	33 (55)	
No	14 (46.7)	13 (43.3)	27 (45)	
Total	30 (100)	30 (100)	60 (100)	
**Age**	58.93 ± 11.12	54.53 ± 9.06	56.73 ±10.3	0.25
**Duration of the disease**	7.63 ± 3.82	6.16 ± 2.13	6.9 ±3.6	0.05

^a^Data are presented in No. (%) or Mean ± SD.

**Table 2.** Independent Samples T-test of Differentials Between Variables Under Study in Case and Control Groups (n = 30)

	Pretest	Posttest		Difference	
Group	Results	Results	P Value ^a^	Results	P Value ^b^ [Confidence interval]
**Self-regulation**					< 0.001 [14.89, 21.77]
Case	112.16 ± 11.03	130.56 ± 1.88	< 0.001	18.40 ± -9.15	
Control	100.83 ± 13.10	100.9 ± 11.24	0.95	0.07 ± -1.86	
**Blood sugar**					< 0.001 [-15.49, -12.56]
Case	206.7 ± 58.78	190.17 ± 54.86	< 0.001	-16.50 ± -3.92	
Control	237.97 ± 47.22	235.5 ± 47.53	0.67	-2.47 ± 0.31	
**Nutrition**					< 0.001 [6.40, 7.27]
Case	9.53 ± 2.55	15.5 ± 1.54	< 0.001	5.97 ± -1.01	
Control	10.33 ± 1.64	9.46 ± 2.27	0.10	-0.87 ± 0.63	
**Physical activity**					< 0.001 [6.59, 7.26]
Case	7.93 ± 1.83	14.13 ± 1.19	< 0.001	6.2 ± -0.64	
Control	10.06 ± 1.98	9.33 ± 1.32	0.09	-0.73 ± -0.66	

^a^ P value: test mean during time with use of paired sample t-test.

^b^ P value: test mean based on differences between the two groups using student's t-test.

As presented in Table 2, there was a significant reduction in the means of blood sugar concerning the case group compared to the control group (P = 0.001). Furthermore, given the means of diet-observing in pretest and posttest, a considerable increase was observed in the case group in comparison with control group (P = 0.001). There is also a dramatic increment in the means of physical activity in pretest rather than posttest of the case group (P = 0.001). Hence, the first, the second and the third hypothesis of the research can be confirmed with a 95% confidence. It is suggested that self-regulated training had positive effect on blood sugar, diet-observing and physical activity in patients with type 2 diabetes. In order to confirm the validity of the results, the means for all the four main variables were evaluated and as it can be seen in Table 2 the differentials were prominent in the case group (second P value).

## 5. Discussion

The objective of the study was to examine the effect of self-regulation learning on controlling type 2 diabetes. To this end, three hypotheses were raised. The results confirmed the first research hypothesis, implying that self-regulated teaching had a significant effect on lowering blood sugar. The finding is in agreement with the results of similar studies conducted in this field (12, 16, 19, 23, 24). Brown is a case in point, affirmed that training programs resulted in substantial improvement in lowering blood sugar in diabetics treated with insulin (20). It seems that self-regulated training has had a positive effect on control of blood sugar in people with diabetes. The results appeared to be satisfactory due to self-regulation training strategies such as self-monitoring (paying regular attention to performance, self-proving, defining the goal and specifying measures to achieve the goal), self-assessment (regular comparison of performance with standards and defined goals), self-reflection (meaningful changes towards achieving the goal) and some other factors such as self-observation, self-reinforcement as well as nutritional and physical activity planning. The outcomes also verified the second research hypothesis, suggesting that self-regulated training had significant effect on diet-observing in people with diabetes. This finding is in compliance with the results of some studies such as Giral Guembe and Esfandyari (17, 21). Esfandyari et al. postulated that there was a significant relation between self-regulation training and nutrition as well as physical activity (21). Moreover, Heydari et al. agreed that empowerment model training led to effective dietary restriction in patients with type 2 diabetes (31).

It seemed that self-regulated teaching had a significant effect on diet-observing in patients of the case group as the result of using self-regulatory learning strategies such as defining goal, dietary planning, receiving help from others, self-monitoring (eating sugar-free foods), preventing overeating and evaluating blood sugar. The results confirmed the third research hypothesis, indicating that self-regulated teaching had significant effect on physical activity in patients with type 2 diabetes. This finding is consistent with the results of similar studies in this field (17, 22, 32). Brown discussed that training programs and planning techniques caused significant improvement in physical activity of diabetics treated with insulin after six months (20). Behncke also believed that self-regulatory exercise and physical activity played an important role in healthy life (33). Given the self-regulatory skills taught and physical activity programs administered to the case group, it was concluded that that training self-regulation strategies helped the patients with diabetes in setting specific goals for having regular physical activity. After assessing their physical activity, patients realized that changing and improving their sedentary behaviors were not only feasible but also had a key role in managing their diabetes.

All in all, it can be concluded that self-regulated teaching has had significant effect on control of type 2 diabetes. Therefore, professionals are recommended to benefit these trainings to help patients with type 2 diabetes. Although the present study has accomplished its goals, a number of caveats need to be noted. In this study only patients with type 2 diabetes were included, self-measuring devices were used as the sources of information and direct instruction method was used as the training strategy. Therefore, future research on nutrition and physical activity would have been more convincing if the researchers have taken the above mentioned issues into account.
